# Patterns of Comorbidities in Psoriasis Patients: A Cross-Sectional Study

**DOI:** 10.7759/cureus.14907

**Published:** 2021-05-08

**Authors:** Rakan S Alajmi, Saeed M Alamoudi, Abdullah A Alabbasi, Alhanouf Alwagdani, Ali A Alraddadi, Awadh Alamri

**Affiliations:** 1 Medicine, King Saud Bin Abdulaziz University for Health Sciences, Jeddah, SAU; 2 Dermatology, King Abdulaziz Medical City, Jeddah, SAU

**Keywords:** psoriasis, comorbidities, patterns

## Abstract

Background: Psoriasis is a chronic, inflammatory, and immune-mediated dermatological disease of unknown etiology with predominant involvement of the skin, nails, and joints. This study aimed to assess comorbidities patterns in psoriasis patients.

Methods: This is a cross-sectional study conducted at King Abdulaziz Medical City (KAMC) in Jeddah, Saudi Arabia. Data were collected through a retrospective chart review of the electronic medical record system (Bestcare, Ezcaretech, Seoul, Korea) and by utilizing a structured data collection sheet.

Results: A total of 128 confirmed psoriasis cases were included with a mean age of 44.2 ± 17.3. The sample had 45.7% females and 54.3% males. Nearly half the patients (46.1%) had no comorbidities, followed by those who had at least one comorbidity (24.2%) and those who had two or more comorbidities (29.7%). Most patients were classified as plaque psoriasis (57.0%), followed by those who had psoriatic arthritis (13.3%). There was no statistical significance between gender, body mass index (BMI), and smoking with the number of comorbidities (P= 0.422, P=0.361, P=0.772); 41.2% of psoriatic arthritis patients and all erythrodermic arthritis patients had two or more comorbidities, which is statistically significant at *p*-value <0.018.

Conclusion: This study demonstrated the prevalence of different comorbidities associated with psoriasis patients; 41.2% of psoriatic arthritis patients and all erythrodermic arthritis patients had two or more comorbidities, which was statistically significant. This necessitates closer monitoring of different comorbidities a psoriasis patient might present with. Especially those who are diagnosed with psoriatic arthritis and erythrodermic arthritis.

## Introduction

Psoriasis is a chronic, inflammatory, and immune-mediated dermatological disease of unknown etiology with predominant involvement of the skin, nails, and joints [1-3]. Psoriasis is diagnosed clinically based on characteristic erythematous, well-demarcated, silvery-white scaly papules or plaques over the extensor aspect of the knees, elbows, and trunk as well as the scalp [1-3]. The pathogenesis is not well understood but is believed to be multifactorial with a pivotal involvement of the immune system, specifically T-cells, after an inciting event in a genetically predisposed individual [1-5]. The global burden of psoriasis is between 1.5%-5.0% but reaches up to 11% in developed countries [1,2,6]. Data about incidence, prevalence, and the clinical profile of patients with psoriasis in the middle east, particularly Saudi Arabia, are lacking.

A rapidly advancing body of knowledge identifies psoriasis as a chronic systemic inflammatory condition that has an increased risk of other comorbid cardiometabolic, gastrointestinal, kidney, and mood disorders [7-11]. Similar to rheumatoid arthritis (RA) and gout, psoriasis is believed to increase risk by multiple mechanisms, most prominent of which is stimulation of inflammatory cells and secretion of proinflammatory cytokines, yet the exact mechanism remains uncertain [7,8]. Moreover, accumulating global evidence has drawn attention to the need of identifying patterns of comorbidities associated with psoriasis to optimize management and enhance our understanding of the underlying pathophysiology [9,12].

Studies on the patterns of comorbidities seen with psoriasis have been explored in numerous countries on various races and ethnicities, but these patterns have only been slightly investigated in the Saudi population [2,10,12,13]. Due to the distinct nature of psoriasis types, classifications, and clinical presentations that separates it from other dermatological conditions, it is essential to investigate patterns of different comorbidities and diseases with psoriasis. Globally, several studies assessed the association between psoriasis and several different comorbidities. However, only a few studies were conducted in Riyadh, and no recent studies in the western region [2,10,13]. In consideration of that, this paper will be of much benefit in improving the standard of practice conducted in our center. In this research, we aim to assess comorbidity patterns in patients with psoriasis treated at King Abdulaziz Medical City (KAMC), Jeddah.

## Materials and methods

This is a cross-sectional study conducted at KAMC in Jeddah, Saudi Arabia. Data were collected through a retrospective chart review of the electronic medical record system (Bestcare, Ezcaretech, Seoul, Korea) and by utilizing a structured data collection sheet. The data collection sheet consisted of socio-demographic variables like age, gender, and body mass index (BMI). Moreover, clinical variables like psoriasis with its different types and comorbidities were assessed.

The obtained data from the collection sheet was entered into Microsoft Excel 2016. Then entered and analyzed in IBM Statistical Package for the Social Sciences (SPSS) version 25.0 (IBM Inc., Chicago, IL). Qualitative variables were presented using descriptive statistics in the form of categories and summarized as frequencies and percentages. Data comparison was interpreted using the chi-square test and Fisher exact test, and a p-value that is less than 0.05 was considered to be significant. All patients’ data is confidential and ethical approval was received from the Institutional Review Board at King Abdullah International Medical Research Centre, National Guard Health Affairs, Jeddah, Saudi Arabia.

## Results

A total of 128 confirmed psoriasis cases were included. Patient ages ranged from 10 to 86 years old (mean ± SD 44.2 ± 17.3). Nearly half the patients (46.1%) had no comorbidities, followed by those who had at least one comorbidity (24.2%) and those who had two or more comorbidities (29.7%). The majority of patients ≤50 years old had no comorbidities, whereas patients >50 years had two or more comorbidities, which is statistically significant at p-value <0.001. The sample had 45.7% females and 54.3% males, showing almost equal female and male distribution. There was no statistical significance (p-value = 0.422) between the distribution of gender and the number of comorbidities. Most patients were nonsmokers (88.3%) and had obese BMI (42.1%) or normal BMI (33.3%). There was no statistical significance between the distribution of BMI or smoking status and the number of comorbidities at p-values <0.361 and <0.772, respectively. The relationship between socio-demographic data and sample comorbidities is shown in Table 1. The highest reported comorbidity was diabetes which was seen in 46 patients, followed by 35 patients with hypertension, whereas the lowest reported comorbidity was inflammatory bowel disease which was seen in only one patient. 

**Table 1:** Relationship between socio-demographic data and sample comorbidities *Chi-Square test **Fisher's exact test

Variables		Comorbidities: n (%)				p-value
		No comorbidities	One Comorbidity	Two or more comorbidities	Total by each row	
Age	≤35	28 (77.8)	8 (22.2)	0	36 (28.1)	0.000**
	36 - 50	26 (57.8)	13 (28.9)	6 (13.3)	45 (35.2)	
	>50	5 (10.6)	10 (21.3)	32 (68.1)	47 (36.7)	
	Total	59 (46.1)	31 (24.2)	38 (29.7)	128 (100)	
Gender	Female	29 (50.0)	15 (25.9)	14 (24.1)	58 (45.7)	0.422*
	Male	29 (42.0)	16 (23.2)	24 (34.8)	69 (54.3)	
	Total	58 (45.7)	31 (24.4)	38 (29.9)	127 (100)	
BMI	Normal	24 (57.1)	7 (16.7)	11 (26.2)	42 (33.3)	0.361*
	Overweight	11 (35.5)	10 (32.3)	10 (32.3)	31 (24.6)	
	Obese	22 (41.5)	14 (26.4)	17 (32.1)	53 (42.1)	
	Total	57 (45.2)	31 (24.6)	38 (30.2)	126 (100)	
Smoking n	No	51 (45.1)	27 (23.9)	35 (31.0)	113 (88.3)	0.772**
	Yes	8 (53.3)	4 (26.7)	3 (20.0)	15 (11.7)	
	Total	59 (46.1)	31 (24.2)	38 (29.7)	128 (100)	

Of the 128 patients that were analyzed, 33.6% had unidentified psoriasis. However, most patients were classified as plaque psoriasis (57.0%), followed by those who had psoriatic arthritis (13.3%). There were very few cases of nail psoriasis (0.8%), pustular psoriasis (1.6%), and inverse psoriasis (1.6%). There were no reported comorbidities among the majority of patients across all types of psoriasis. However, a proportion of psoriatic arthritis (41.2%) and all erythrodermic arthritis (100%) patients had two or more comorbidities, which was statistically significant at p-value <0.018. The relationship between psoriasis classifications and sample comorbidities is shown in Table 2.

**Table 2:** Relationship between the classification of psoriasis and sample comorbidities **Fisher's exact test

		Comorbidities: n (%)					
Variables		No comorbidities	One Comorbidity	Two or more comorbidities	Total by each row		p-value
Psoriasis Classification	Psoriatic arthritis	6 (35.3)	4 (23.5)	7 (41.2)	17 (13.3)		0.018**
	Inverse psoriasis	0	1 (50.0)	1 (50.0)	2 (1.6)		
	Plaque psoriasis	30 (55.6)	13 (24.1)	11 (20.4)	54 (42.2)		
	Erythrodermic psoriasis	0	0	3 (100)	3 (2.3)		
	Guttate psoriasis	6 (100)	0	0	6 (4.7)		
	Nail Psoriasis	0	1 (100)	0	1 (0.8)		
	Pustular psoriasis	0	1 (50.0)	1 (50.0)	2 (1.6)		
	Unidentified	17 (39.5)	11 (25.6)	15 (34.9)	43 (33.6)		
	Total	59 (46.1)	31 (24.2)	38 (29.7)	128 (100)		

## Discussion

This study aimed to assess psoriasis types and identify comorbidities patterns in psoriasis patients treated at KAMC. In this study, male to female ratio was almost equal. On the contrary, studies conducted in India showed that psoriasis was more common among male patients [1]. Moreover, male predominance was seen in a study conducted in Nigeria [2]. Furthermore, the same Nigerian study reported that the majority of patients were seen in their fourth decade [2]. This was mirrored in a recent study conducted in India which showed that the majority of their study’s sample belonged to the fourth decade [3]. In our study, only 35.2% of the patients were between 36 and 50 years old.

The study at hand found that chronic plaque psoriasis was the most common phenotype followed by unidentified psoriasis and psoriatic arthritis while nail psoriasis was the least common phenotype. These findings are in line with other studies in the literature as Bedi found that chronic plaque psoriasis is the most common type among their sample of 530 subjects accounting for 90% [1]. Another study in Nigeria also demonstrated that plaque psoriasis was the most common manifestation representing 66.1% of the sample [2]. In addition, studies in Thailand have shown results aligned with ours as plaque psoriasis was the most common presentation accounting for 72.8%. Accumulating evidence suggests that chronic plaque psoriasis (vulgaris) is the most common phenotype regardless of geographic and ethnic boundaries.

Only a handful of our sample demonstrated guttate psoriasis representing 4.7% of the total sample. These findings are consistent with reports of low proportion of guttate psoriasis from India where they found that only 4% demonstrated guttate psoriasis among their population [3]. Moreover, previous local studies show that guttate psoriasis is among the least common phenotypes of psoriasis in our population accounting for 1.9% in the eastern region of Saudi Arabia [4] Jiamton et al. along with Ayanlowo and Akinkugbe show contrasting results to ours as guttate psoriasis was the second most common phenotype and the third most common phenotype accounting for 22.7% and 27.4% of their samples, respectively [2,5].

Inverse and pustular psoriasis were among the least common phenotypes of psoriasis across different studies, including the study at hand. Whereas our study demonstrated isolated nail psoriasis as the least common phenotype, an African study illustrated that nail psoriasis was the third most common manifestation accounting for up to 29% of patients [2]. Additionally, Bedi found that isolated nail psoriasis occurred in only 6% of patients whereas it can be found in a total of 74% of psoriatic patients in conjunction with other types [1]. Scarpa et al. found that nail psoriasis was found more commonly in patients with psoriatic arthritis and further studies showed that the relationship between nail psoriasis and psoriatic arthritis reaches statistical significance; however, no specific nail abnormality was linked to developing arthritic psoriasis [5,6].

In this study, more than half of the subjects had reported at least one comorbidity; hence, the prevalence of comorbidity in the sample is 69 out of 128 (53.9%) subjects. This finding was in alignment with a study conducted by Wu et al. which reported a comorbidity prevalence of 39% among their sample [7]. Moreover, 31 (24%) of our subjects reported only one comorbidity and 31 (29.7%) reported two or more, while the subjects who did not have comorbidities composed 59 (46.1%) of participants. In contrast, a study conducted by Sultana et al. described that 11 (7.33%) of their psoriasis patients had one comorbidity and five (3.33%) of them reported multiple comorbidities [8]. It was noted in this study that patients with psoriatic arthritis (41.2%) and erythrodermic arthritis (100%) appeared to have an increased incidence of having two or more comorbidities compared to other psoriatic subtypes, having statistical significance at p-value <0.018.

Comorbidities classically associated with psoriasis are psoriatic arthritis, Crohn's disease, psychological/psychiatric disorders, and uveitis [9]. In our study, psoriatic arthritis was found in 17 (13.28%) of the sample, while psychological/psychiatric disorders were the least reported comorbidities as they were reported by only two (2.56%) subjects. In recent years, metabolic syndrome as a whole and its components have been associated with psoriasis [9]. In our study, the highest reported comorbidities were diabetes found in 46 (35.94%) patients, followed by 35 (27.34%) patients with hypertension and 22 (17.19%) patients with dyslipidemia. These findings are consistent with a study conducted by Hassan et al. in 2017, which reported that the most reported comorbidities in patients with psoriasis were diabetes mellitus 53 (65%) followed by dyslipidemia 50 (62%) and hypertension 39 (48%) [10].

Recent studies also showed an increased prevalence of celiac disease, nonalcoholic fatty liver disease, and erectile dysfunction in patients who have psoriasis [9]. In our study, eight (6.25%) subjects reported liver disease, and this finding might be related to the medication side-effects used by the psoriatic patient to control their lipid profile. In contrast to the literature [7-11], in this sample, there were no reports of Crohn’s disease, and the only gastrointestinal disease was inflammatory bowel disease which was the least reported comorbidity 2 (2.56%). Regarding other comorbidities, a study in Afula by Zoabi et al. showed that hypothyroidism was found in 5% of psoriatic patients while it was 10.94% in this study [11].

There are some limitations in our study. The reporting of comorbidities in psoriasis patients was based on the patient’s history and available medical records. No active screening or confirmatory investigations for these conditions were performed. Hence, over-or underestimation of the prevalence of comorbidities may occur. Additionally, the records from which data was collected had some missing information like the severity of psoriasis (psoriasis area and severity index score).

## Conclusions

This study presented the prevalence of different comorbidities associated with psoriasis patients. The study showed that 53.9% of psoriasis patients had at least one comorbidity. There was no statistical significance between the gender, BMI, and smoking with the number of comorbidities (P= 0.422, P=0.361, P=0.772); 41.2% of psoriatic arthritis patients and all erythrodermic arthritis patients had two or more comorbidities, which is statistically significant at p-value <0.018. This necessitates closer monitoring of different comorbidities a psoriasis patient might present with, especially those who are diagnosed with psoriatic arthritis and erythrodermic arthritis. Moreover, larger multi-center studies should be conducted in the future.
